# Supplementary figures and images for: Tryptophan-kynurenine pathway: a possible new mechanism for the prevention and treatment of reproductive system-related diseases
Source: PeerJ. 2025 Nov 17;13:e20342. doi: 10.7717/peerj.20342 (PMC12633197; doi:10.7717/peerj.20342)

## Identification of studies via databases/registers

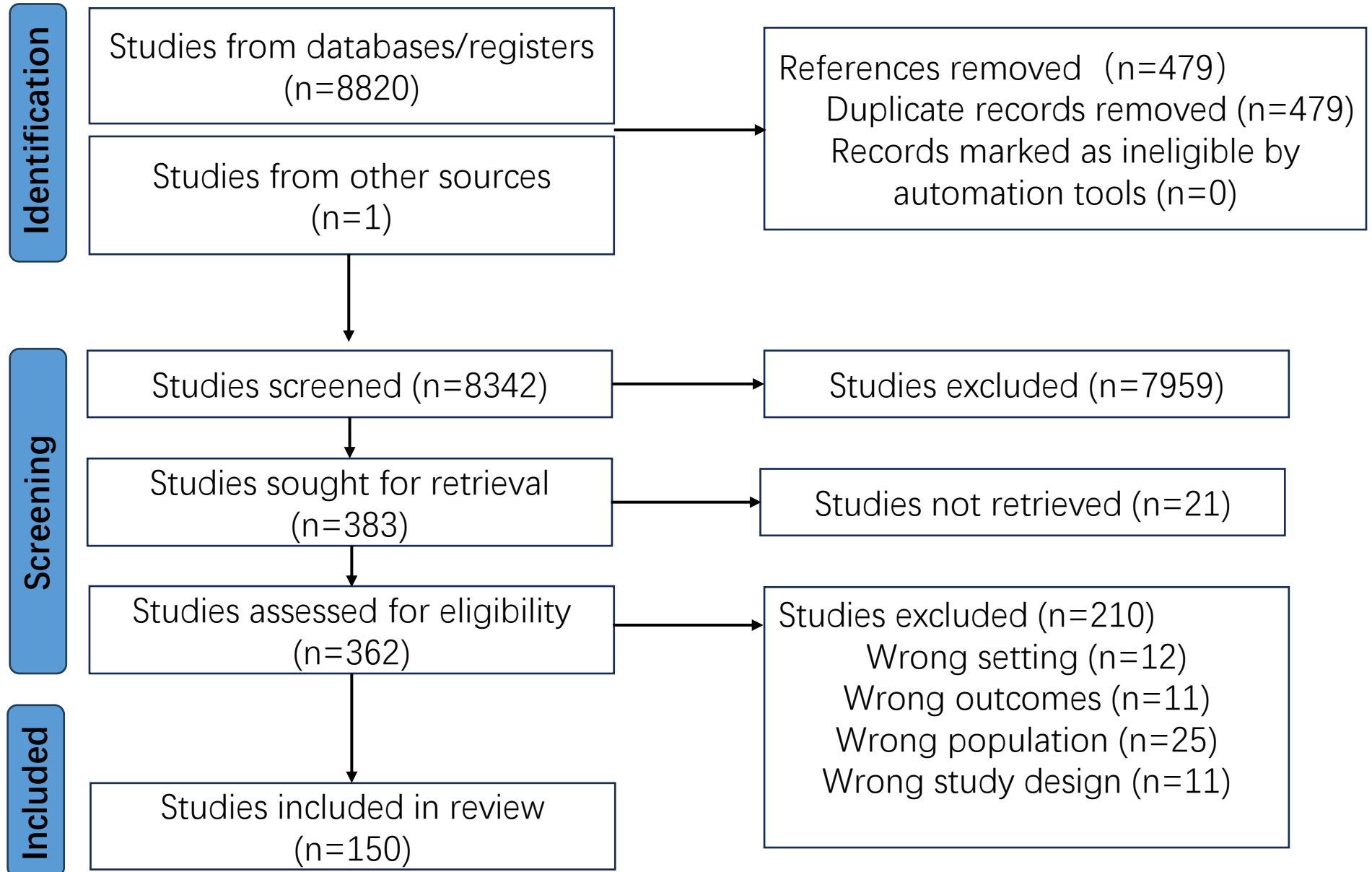

Supplement: Supplemental Information 1 [file peerj-13-20342-s001.pdf]
